# Oxidative Stress and Advanced Lipoxidation and Glycation End Products (ALEs and AGEs) in Aging and Age-Related Diseases

**DOI:** 10.1155/2019/3085756

**Published:** 2019-08-14

**Authors:** Nurbubu T. Moldogazieva, Innokenty M. Mokhosoev, Tatiana I. Mel'nikova, Yuri B. Porozov, Alexander A. Terentiev

**Affiliations:** ^1^I.M. Sechenov First Moscow State Medical University (Sechenov University), 8 Trubetskaya Street, Moscow, 119991, Russia; ^2^N.I. Pirogov Russian National Research Medical University, 1 Ostrovityanov Street, Moscow, 117997, Russia; ^3^Saint Petersburg National Research University of Information Technologies, Mechanics and Optics, 49 Kronverksky Prospect, St. Petersburg, 197101, Russia

## Abstract

Oxidative stress is a consequence of the use of oxygen in aerobic respiration by living organisms and is denoted as a persistent condition of an imbalance between the generation of reactive oxygen species (ROS) and the ability of the endogenous antioxidant system (AOS) to detoxify them. The oxidative stress theory has been confirmed in many animal studies, which demonstrated that the maintenance of cellular homeostasis and biomolecular stability and integrity is crucial for cellular longevity and successful aging. Mitochondrial dysfunction, impaired protein homeostasis (proteostasis) network, alteration in the activities of transcription factors such as Nrf2 and NF-*κ*B, and disturbances in the protein quality control machinery that includes molecular chaperones, ubiquitin-proteasome system (UPS), and autophagy/lysosome pathway have been observed during aging and age-related chronic diseases. The accumulation of ROS under oxidative stress conditions results in the induction of lipid peroxidation and glycoxidation reactions, which leads to the elevated endogenous production of reactive aldehydes and their derivatives such as glyoxal, methylglyoxal (MG), malonic dialdehyde (MDA), and 4-hydroxy-2-nonenal (HNE) giving rise to advanced lipoxidation and glycation end products (ALEs and AGEs, respectively). Both ALEs and AGEs play key roles in cellular response to oxidative stress stimuli through the regulation of a variety of cell signaling pathways. However, elevated ALE and AGE production leads to protein cross-linking and aggregation resulting in an alteration in cell signaling and functioning which causes cell damage and death. This is implicated in aging and various age-related chronic pathologies such as inflammation, neurodegenerative diseases, atherosclerosis, and vascular complications of diabetes mellitus. In the present review, we discuss experimental data evidencing the impairment in cellular functions caused by AGE/ALE accumulation under oxidative stress conditions. We focused on the implications of ALEs/AGEs in aging and age-related diseases to demonstrate that the identification of cellular dysfunctions involved in disease initiation and progression can serve as a basis for the discovery of relevant therapeutic agents.

## 1. Introduction

Living cells produce various kinds of reactive oxygen species (ROS) such as superoxide anion radical (O_2_^•−^), hydrogen peroxide (H_2_O_2_), and hydroxyl radical (HO^•^) [1, 2]. The major endogenous sources of ROS include mitochondrial electron-transportation chain (ETC) complexes I and III and the NADPH oxidases of NOX family enzymes [3–5]. Additionally, ROS may be produced by xanthine oxidase, cyclooxygenases (COXs, prostaglandin G/H synthases), lipoxygenases, and the cytochrome P450- (CYP-) containing monooxygenase system [6–9]. ROS generation may be induced by exogenous factors such as UV light, X-ray, and *γ*-ray irradiations, air pollutants, tobacco smoke, heavy metals, and certain drugs [10, 11].

Under physiological conditions, living cells maintain low intracellular concentrations of ROS due to the activity of the endogenous antioxidant system (AOS) composed of both enzymatic and nonenzymatic components capable of ROS scavenging and, thereby, protecting cells from the deleterious effects of high ROS concentrations (reviewed in [12–14]). The enzymatic components include superoxide dismutases (SODs), catalase, peroxiredoxins (Prxs), glutathione peroxidases (GPx), and glutathione reductase (GR), while nonenzymatic components include ascorbic acid, alpha-tocopherol, retinol, and various molecules with thiol groups such as glutathione, lipoic acid, small protein thioredoxin, as well as transition-metal ions such as Fe, Cu, Zn, and Mn [15–20].

At low concentrations, ROS exert regulatory effects on cellular functions including proliferation, differentiation, migration, and survival [21, 22]. This is provided by their involvement in reversible posttranslational modifications of key redox-sensitive amino acid residues in enzymes, intracellular effectors of signal transduction pathways (protein kinases and protein phosphatases), transcription factors, cytoskeletal proteins, and molecular chaperones [23–26]. Through this, oxidative protein modifications may be caused either directly by ROS themselves or indirectly by secondary products of ROS-induced oxidation reactions occurring on both protein backbone and amino acid side chains [27, 28].

However, insufficient AOS activity causes the accumulation of ROS, which leads to oxidative stress that is denoted as a persistent condition of an imbalance between ROS generation and the ability of a biological system to detoxify them leading to disruption in redox signaling/control and/or molecular damage [29]. Oxidative stress induces lipid peroxidation and glycoxidation reactions, which lead to the formation of highly reactive and electrophilic compounds that attack free amino groups in proteins causing their covalent modifications and resulting in the generation of advanced lipoxidation end products (ALEs) and advanced glycation end products (AGEs) [30].

ALE and AGE formation causes an impairment in the protein structure due to covalent cross-linking resulting in protein oligomerization and aggregation. This leads to alterations in cellular functions, cell damage, and death. For example, the impairment in mitochondrial, endoplasmic reticulum (ER), and extracellular matrix (ECM) proteins and those involved in cell cycle and control of gene expression has been observed in various studies [31, 32]. Oxidative stress and oxidative stress-induced ALE and AGE formation have been implicated in aging and in a variety of age-related chronic diseases [33–37].

The present review focuses on recent advancements in investigating the consequences of oxidative stress-induced ALE/AGE accumulation for cellular functions and the implication of ALE/AGE formation in aging and age-related human diseases such as chronic/acute inflammation, neurodegenerative disorders, atherosclerosis, and vascular complications of diabetes mellitus (DM).

## 2. Implication of ALEs and AGEs in Aging

### 2.1. Oxidative Stress and Aging

Aging is a progressive time-dependent functional decline in an organism's physiological integrity and adaptability followed by a consequent irreversible decrease in its fertility and an increase in morbidity and mortality risk [38]. In 1959, Denham Harman postulated a free radical theory of aging that points to ROS accumulation as the underlying reason for biomolecular oxidation and cellular damage and as the explanation for the alterations in cellular functions during aging [39]. Since that time, the oxidative stress theory of aging has gained considerable acceptance, despite numerous other proposed theories on biological aging and senescence [40].

The reduction of oxidative stress by ROS scavengers followed by the delay of the age-associated decline in physiological processes and marked prolongation in the mean lifespan can be considered as a confirmation of the oxidative stress theory of aging [41]. This theory has also been approved in many animal models including *S. cerevisiae*, transgenic mice, and long-lived species such as *C. elegans*, birds, and naked mole-rat [42–44]. Among them, the naked mole-rat (NMR, *Heterocephalus glaber*) is the longest-living rodent known with a maximum lifespan of more than 28.3 years, which is 9 times longer than that of similar-sized laboratory mice. Body composition, physiological functions including reproductive function, and tissue morphology of NMRs can be maintained from 2 to 24 years almost with no changes and showing negligible senescence and no spontaneous neoplasm [45].

Perez et al. [46] showed that the amount and activities of both ROS and the antioxidant system in NMRs are similar to those of shorter-living mice; however, NMRs exhibited higher levels of oxidative biomolecular damage (DNA damage, lipid peroxidation, and protein carbonyl formation) even at an early age. However, NMRs demonstrated a higher amount of free thiol groups and lower levels of urea-induced protein unfolding and protein ubiquitination as well as higher proteasome activity as compared to young C57BL/6 mice [46]. Interestingly, no one of these parameters was significantly altered during the two decades of the NMRs' lifespan. These data indicate that the existence of molecular mechanisms underlying the maintenance of cellular homeostasis and biomolecular stability and integrity are crucial for cellular longevity and successful aging.

Indeed, alterations in the structure, functions, and oxidation state of muscle proteins have been observed in aged F344BN mice [47]. The content of regulatory proteins was reduced by up to 75%, while the catalytic activity of enzymes decreased by up to 50% in mice with aging. Additionally, Duchenne muscular atrophy and loss of nerve supply along with increased expression of immunoproteasome subunits have been observed in aged animals [48]. The increased lifespan of Tq mice has been reported to associate with the stimulation of mitogen-activated protein kinase- (MAPK-) mediated redox signaling, the increased expression of stress-protective heat shock protein 25 (HSP25), and the activation of antioxidant enzymes, catalase, and SODs, suggesting that the oxidative stress-induced stimulation of endogenous defense mechanisms plays key roles in providing health and longevity [49].

Proteostasis is an overall cellular protein homeostasis provided by integrated protein control quality pathways including biosynthesis, folding, trafficking, and elimination/degradation of damaged proteins [50, 51]. Maintaining proteostasis is an important component of successful aging because, in most metazoans, aging has been shown to be accompanied by a decline in the activities of the protein quality control machinery that includes molecular chaperones, ubiquitin-proteasome system (UPS), and autophagy/lysosome activity, which results in the accumulation of damaged and self-aggregating proteins [52]. During aging, oxidative protein damage and covalent cross-linking followed by the accumulation of the so-called “aggresomes” that are toxic for cells have been shown [53, 54]. Long-lived species have been observed to possess improved proteostasis in comparison with short-lived species as assessed by elevated HSP levels, enhanced macroautophagy, and the UPS activity [55]. Additionally, the reestablishment of proteostasis due to lysosome activation followed by a metabolic shift that mobilizes the degradation of protein aggregates has been observed in immortal *C. elegans* germ lineages [43].

One of the most studied features of aging is the manifestation of mitochondrial dysfunction [56, 57]. Mitochondria are considered as both a major site of ROS generation and the main target for ROS attack. The age-related increase in mitochondrial ROS production by complex I, oxidative stress-induced mutations in mitochondrial DNA (mtDNA), and accumulation of mtDNA fragments inside the nucleus have been observed in mouse liver [58]. These changes were accompanied by oxidative damage and lipoxidation of mitochondrial proteins including enzymatic components of ETC and accumulation of lipofuscin produced by covalently cross-linked and aggregated proteins; all these alterations were abolished by rapamycin treatment. Additionally, a lesser amount mitochondrial ROS production and higher cardiolipin content in erythrocytes of long-lived species as compared to short-lived ones have been reported [44]. Thus, age-dependent accumulation of oxidized proteins may be caused by both an increase in mitochondrial ROS production and a decline in the proteolytic capacity of either the ubiquitin/proteasomal or lysosomal pathway [59].

The Nrf2 transcription factor serves as a master regulator of cell response to oxidative stress, the Nrf2 dysfunction being observed in various cell types during aging. The overexpression of Nrf2 target genes, NADPH quinone oxidoreductase 1 (NQO1), glutamate cysteine ligase (GCLM), and heme oxygenase 1 (HO1) have been shown in aged mouse retinal pigment epithelium (RPE) cells as compared to younger mice under oxidative stress conditions [60]. Old mice also exhibited higher O_2_^•−^ and MDA levels than younger mice. The same genes were overexpressed under Nrf2 induction conditions in the bronchial epithelium cells of old humans as compared to young adult persons [61]. A disruption in Nrf2 signaling causes reduced cell migration and an impaired ability of the coronary artery endothelial cells to form capillary-like structures [62].

The antioxidant system, including the glutathione (GSH/GSSG) system and SODs, has been shown to be involved in successful aging through the maintenance of intracellular redox balance. Indeed, the altered ratio between reduced, GSH, and oxidized, GSSG, forms of glutathione in aging has been demonstrated by measurements of GSH concentration in red blood cells and levels of plasma oxidative stress biomarkers such as F2-isoprostanes in younger and elderly persons [63]. The elderly persons had markedly lower concentrations of glycine, cysteine, and GSH along with decreased GSH biosynthesis in erythrocytes as compared to those in younger persons. However, glycine and cysteine supplementation led to an increase in GSH concentration and rate of its biosynthesis along with a significant decrease in levels of oxidative stress biomarkers in the blood plasma.

A reduced ROS level due to the activation of another AOS component, Mn-superoxide dismutase (SOD2), through its deacetylation at the evolutionarily conserved Lys122 residue by the conserved family of NAD^+^-dependent deacetylases, sirtuins, have been reported as a factor involved in lifespan control [64, 65]. Mammalian sirtuins 1 and 3, SIRT1 and SIRT3, have been shown to regulate the activity of SOD2 to protect muscle cells from oxidative stress [66]. They can promote mitochondrial biogenesis by activating PGC-1*α* that is a transcriptional coactivator upregulating antioxidant enzymes such as GPx, catalase, and SOD2 [67].

### 2.2. ALEs and AGEs in Aging

Oxidative stress induces endogenous formation and accumulation of both ALEs and AGEs, which can be produced from the same precursors such as glyoxal and methylglyoxal and through the same intermediates such as *N*-(carboxymethyl)-lysine (CML) and *N*-(carboxymethyl)-cysteine (CMC). ALEs are generated due to lipid peroxidation reactions, while AGEs result from glycoxidation reactions; both of the pathways give rise to an extraordinarily complex mixture of interrelated compounds [30]. These compounds include highly reactive electrophilic aldehydes and their derivatives such as 4-hydroxy-2-nonenal (HNE), 4-oxo-2-nonenal (ONE), 4-hydroxy-hexanal (HHE), acrolein (ACR), and malonic dialdehyde (MDA) [68, 69]. They interact with free amino groups in protein to cause their covalent modification, cross-linking, oligomerization, and aggregation. These processes cause intracellular damage, impaired cell functions, and, ultimately, cell death to be implicated in aging and various age-related chronic diseases [70, 71].

#### 2.2.1. Roles of ALEs in Aging

Changes in the amount of lipid peroxidation products and activities of COX-2 and CYP2JA in human brain have been reported to occur in an age-dependent manner [72]. A significant increase in lipid peroxidation and oxidative protein modification levels accompanied by the loss of thiol groups, accumulation of dityrosine, and ALE formation has been observed in mitochondria and synaptosomes during brain aging in rats [73]. Interestingly, the higher membrane resistance to lipid peroxidation and the lower molecular damage caused by protein lipoxidation have been shown to associate with significantly reduced desaturase activity and peroxisomal betaoxidation in the brain and spleen of exceptionally old (128 ± 4 weeks) and adult (28 ± 4 weeks) female mice as compared to old (76 ± 4 weeks) animals [74].

Aldehydes generated from polyunsaturated fatty acid (PUFA) peroxidation such as HNE, MDA, and ACR have been shown to form protein adducts that accumulate in the intima, media, and adventitia layers of the human aorta leading to progressive cellular dysfunction and contributing to the process of aging [75]. HNE, the most reactive and abundant endogenously generated *α*,*β*-unsaturated hydroxyl-aldehyde, has been shown to contribute to inhibiting elastin repair by antagonizing elastogenic signaling of transforming growth factor-*β* (TGF-*β*) through the inhibition of Smad2 translocation into the nucleus of human and murine skin fibroblasts [76].

Additionally, the accumulation of HNE-modified adducts, the decrease in elastin content, and the modification of the epidermal growth factor (EGF) receptor by NHE have been observed in the aorta of aged C57BL/6 mice. The content of elastin in connective tissue decreased, and the structure of elastin fibers was significantly altered with aging; however, the oxidative protein modification level was very poor indicating a complex role of ALEs in vascular wall remodeling during aging [76].

#### 2.2.2. Ages and AGE-RAGE Axis in Aging

AGE manifestation, especially in connective tissue, which leads to age-dependent damage and covalent cross-linking in ECM adhesion proteins such as collagen, laminin, and elastin has been shown to contribute to the loss of skin and vessel elasticity and degeneration of cartilages, ligaments, and eye lens [77, 78]. The accumulation of AGEs and the fluorescent age pigment, lipofuscin, both of which are typically of brown color, has been shown to associate with aging and age-related chronic diseases contributed by age-dependent inhibition of both proteasomal and lysosomal protein degradation pathways [79, 80].

Various age-related diseases arise due to alterations in cell signaling pathways that proceed with the involvement of the receptors for AGEs (RAGEs) and the AGE-RAGE axis. For example, the colocalization of CML and RAGE along with the activation of nuclear factor-*κ*B (NF-*κ*B) has been observed in patients with age-related macular degeneration indicating the possible role of the AGE-RAGE axis and the NF-*κ*B transcription factor in the pathogenesis of the disease [81]. The accumulation of both AGEs and RAGEs in RPE and photoreceptor cells has been accompanied by NF-*κ*B nuclear translocation and cell apoptosis [82]. These data allowed suggesting that AGE accumulation induces RPE/photoreceptor cell activation during normal aging and contributes to age-related pathologies in human retinas.

Additionally, diet-derived AGEs and lipofuscin have been reported to disrupt the overall protein homeostasis and to reduce the lifespan of *D. melanogaster* [83]. Oral administration of glucose-, fructose-, and ribose-modified albumin or artificial lipofuscin caused the accumulation of AGEs in fly somatic tissues and hemolymph, and this was accompanied by oxidative stress and the upregulation of lysosomal cathepsin B activity. Interestingly, the decreased glucose level observed under caloric restriction with no malnutrition conditions led to the inhibition of enzyme activities and the decrease in concentrations of metabolites of the polyol pathway, sorbitol and fructose. This contributed to the beneficial effects of caloric restriction including the increase in the NADPH level required for other reduction reactions such as GSH and other forms of AOS component regeneration, and counteracted age-related changes derived from the activities of the polyol pathway [84].

Thus, experimental data evidence key roles of both ALEs and AGEs in the process of aging, being considered as biomarkers of oxidative stress and mitochondrial dysfunction and as factors of aging and age-associated chronic pathologies [85].

## 3. Roles of ALEs and AGEs in Age-Related Chronic Diseases

### 3.1. Neurodegenerative Diseases

Oxidative stress and oxidative protein damage can accelerate the formation of toxic protein oligomers and aggregates in the nucleus and cytoplasm of nerve cells, which contributes to the pathogenesis of neurodegenerative diseases such as Alzheimer's disease (AD), Parkinson's disease (PD), Huntington's disease (HD), and amyotrophic lateral sclerosis (ALS) [86–88]. Despite their distinct causative factors and clinical symptoms, these diseases have common pathogenetic features such as mitochondrial dysfunction and ER stress implicated in excessive ROS accumulation, impairment in proteostasis network, and neuroinflammation [89].

**Figure 1:**
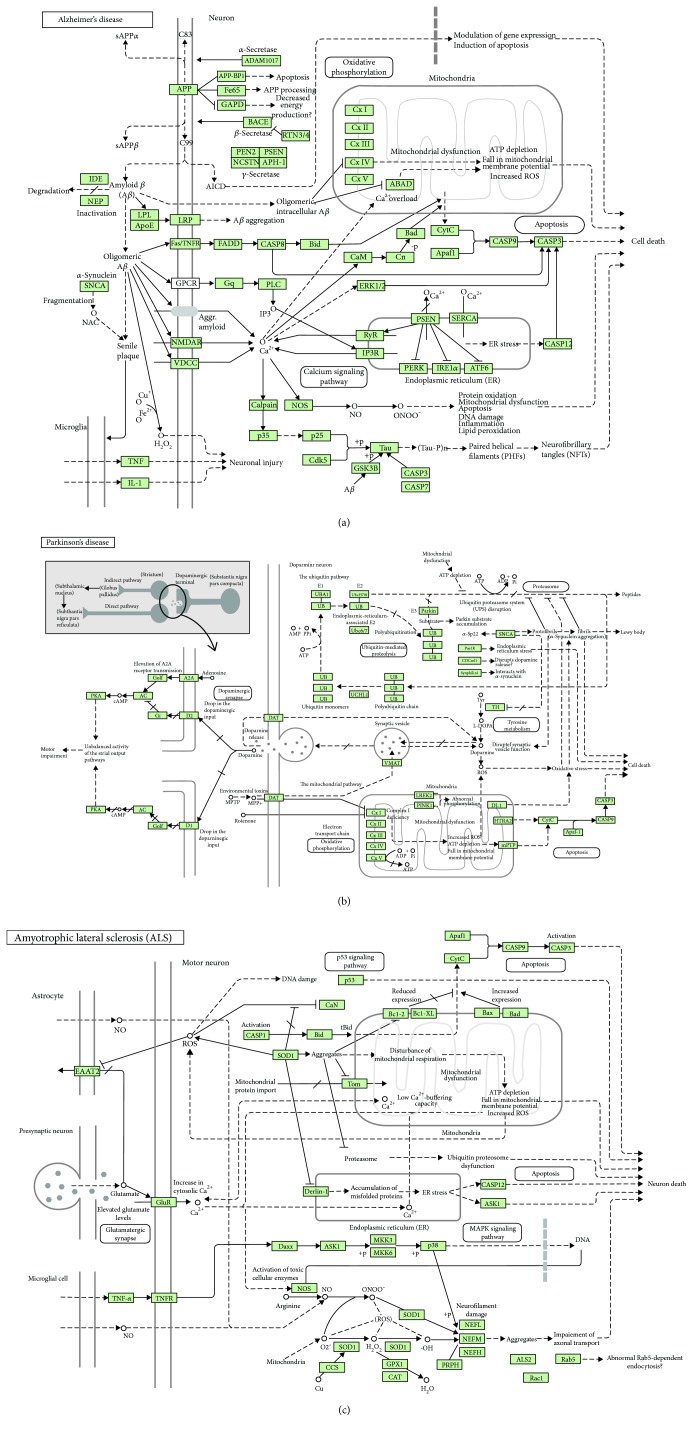
Schematic KEGG map representations of signaling pathways involved in Alzheimer's disease (a), Parkinson's disease (b), and amyotrophic lateral sclerosis (c). Oxidative stress-induced alterations in signaling pathways, which cause mitochondrial dysfunction, endoplasmic reticulum (ER) stress, and dysregulation of the ubiquitin-proteasome system (UPS) and the autophagy/lysosomal protein quality control machinery, followed by neuronal death, are also shown. Here, → indicates stimulating effects and **—** indicates inhibitory effects. (a) AD is characterized by the formation of amyloid precursor protein-derived amyloid *β*-peptide (A*β*), a major component of senile plaques, which forms oligomers to induce pathways initiated by the following receptors: (i) LRP, an apoE receptor; (ii) amyloid precursor protein (APP), an integral membrane protein, mutations of which cause susceptibility to familial AD; (iii) TNF-*α* receptor (Fas/TNFR) to activate caspases; (iv) GNAQ (Gq)/G-protein-coupled receptor (GPCR) to stimulate phospholipid C (PLC) followed by the activation of inositol-3-phosphate receptor (IP_3_R) and ER stress; (v) *N*-methyl-D-aspartate receptor (NMDAR) to cause hyperphosphorylation of tau receptors, and (vi) voltage-gated (dependent) calcium channels (VDCC) followed by neuronal damage through mitochondrial dysfunction and disruption of calcium release from ER. Presenilin 1 and 2 (PSEN1 and PSEN2) proteins belong to *γ*-secretases that generate A*β*. (b) PD results from the death of dopaminergic neurons in the substantia nigra pars compacta (SNs). Normally, dopamine active transporter (DAT) pumps dopamine out of the synaptic clefts into the cytoplasm. The early onset of PD is associated with mutations in synuclein-alpha (SNCA), ubiquitin carboxy-terminal hydrolase L1 (UCHL1), PTEN-induced kinase 1 (PINK1), leucine-rich repeat kinase 2 (LRRK2), mitochondrial serine protease 2 (HTRA2), parkin, and parkin-associated protein DJ1 involved in oxidative stress. (c) ALS is a lethal disorder characterized by the death of motor neurons in the brain and spinal cord. Mutations in SOD1 may interfere with the neurofilament heavy polypeptide (NEFH) and the translocation machinery, the translocase of the inner/outer membrane (TIM/TOM) that is involved in familial ALS. Proapoptotic THF*α* acts through its receptor, TNFR, to induce inflammation and apoptotic cell death. The main glutamate transporter protein, excitatory amino acid transporter (EAAT2), is inhibited by ROS produced by mitochondria. Glutamate acts through its receptor (GluR) to increase calcium release from ER and to enhance oxidative stress and mitochondrial damage. Permission 190019 for usage of the following KEGG pathway images was kindly granted by Kanehisa Laboratories [141]: map05010—Alzheimer's disease; map05012—Parkinson's disease; map05014—amyotrophic lateral sclerosis (ALS).

Normal aging and neurodegeneration can be distinguished by the measurement of AGE concentration in the brain tissue and cerebrospinal fluid. AGE/RAGE manifestation indicates neuropathological and biochemical alterations such as excessive protein cross-linking, inflammation, and neuronal cell death. For example, the accelerated accumulation of AGEs in pathological deposits such as amyloid fibrils and senile plaques has been observed in AD (Figure 1(a)), the most common age-related dementing disorder [90]. The measurement of various AGEs and ALEs in the brain cortex of AD patients demonstrated a significant, although heterogeneous increase in the concentrations of CML, *N*(epsilon)-malondialdehyde-lysine, *N*(epsilon)-carboxyethyl-lysine, and other protein oxidation adducts [91]. Methylglyoxal has been suggested to be one of the major carbonyl species responsible for AGE formation in AD [92].

AGEs can stimulate the expression of inducible nitric oxide synthase (iNOS), and colocalization of AGEs and iNOS has been demonstrated in astrocytes and microglia of AD patients (Figure 1(a)) as revealed by immunochemical analysis [93]. Additionally, an increase in traumatic brain injury-induced nitric oxide generation catalyzed by iNOS and persistent tyrosine nitration adjacent to the injury site have been reported [94]. These effects were accompanied by oxidative stress-induced cell death through apoptosis induction and receptor-mediated serine/threonine protein kinase-mediated necrosis.

Mitochondrial dysfunction and mutations in mtDNA genes encoding ETC complex I subunits with the subsequent impairment in ATP production and elevated ROS generation along with disruption in both UPS and autophagy-lysosome protein degradation pathways have been observed in all types of neurodegeneration [95]. Damaged mitochondria accumulate tensin homolog deleted from chromosome 10- (PTEN-) induced kinase 1 (PINK1) that recruits parkin, a protein of the ubiquitin E3 ligase complex, as shown in PD patients (Figure 1(b)) [96, 97]. This causes the ubiquitination of mitochondrial proteins, which can further bind to the autophagic proteins, p62/SQSTM1 and lc3, resulting in the degradation of mitochondria through the autophagy pathway, the process denoted as mitophagy [98]. Significant increases in the expression of p62/SQSTM1 both at the mRNA and protein levels along with the activation of mitochondrial/lysosomal biogenesis following PINK1/parkin-mediated mitophagy have been observed in familial AD [99].

Nrf2 and transcription factor EB (TFEB), which play key roles in mitochondrial and lysosomal biogenesis, respectively, have been demonstrated to translocate into the nucleus following the mitophagy induction. Additionally, the multifaceted protective potential of Nrf2 signaling in patients with neurodegenerative diseases and in primary mouse HD and WT microglia and astrocytes has been reported [100, 101]. Oxidative stress-induced covalent modification of Cys151 in Kelch-like ECH-associated protein 1 (Keap1), the E3 ligase substrate adaptor protein and primary negative regulator of Nrf2, has been shown in HD [100]. Nrf2 expression is orchestrated and amplified by the coexpression of antioxidant and anti-inflammatory genes as shown, for example, in the primary monocytes from HD patients, in which the repressed expression of proinflammatory cytokines such as IL-1, IL-6, IL-8, and tumor necrosis factor-*α* (TNF-*α*) was observed (Figure 1(a)).

The oxidative modification of Cys111 in Cu/Zn SOD (SOD1) has been implicated in the pathogenesis of various diseases, while mutation in SOD1 (Figure 1(c)) has been found in 20% of familial ALS [102]. Unlike native SOD1, cysteinylated SOD1 is not oxidized, suggesting that the cysteinylation protects this antioxidant enzyme from hydrogen peroxide-induced oxidation as shown in the culture of nerve cells. The existence of the cross-talk between the overexpression of SOD1 and regulation of mitochondrial unfolded protein response (UPR) has been postulated [103].

In the nervous system, proteasomes play key roles in maintaining the neuronal protein homeostasis, while an alteration in their activity contributes to pathogenesis of neurodegenerative diseases [104, 105]. The accumulation of large-ordered fibrils formed by *β*-sheet-enriched proteins denoted as amyloid fibrils in neuronal cells is characteristic for all types of neurodegenerative diseases, being a result of UPS dysfunction and, consequently, accumulation of polyubiquitinylated proteins in nervous tissue [106, 107]. A decreased capacity for the removal of oxidized proteins and the accumulation of damaged and misfolded proteins causes metabolic dysfunction and initiates cell death through apoptosis or necrosis. These disturbances lead to progressive amyloid plaque formation, loss of neurons, brain atrophy, cerebrovascular amyloid angiopathy, and vascular mineralization in an age-dependent manner [108].

ER stress has also been implicated in many chronic neurodegenerative diseases including AD and HD, while prolonging ER stress results in cell death. An important role in this process belongs to ER-localized stress-sensing and stress-triggering proteins such as IRE1*α*, ATF6, and PERK (Figure 1). During UPR, they activate the apoptotic signaling pathway, while fortilin, a prosurvival molecule, inhibits apoptosis by directly binding to IRE1*α* and reducing both its kinase and RNAse activities [109].

### 3.2. Atherosclerosis

The accumulation of both ALEs and AGEs progressively leads to cellular dysfunction and tissue damage involved in the progression of other oxidative stress-induced chronic diseases such as atherosclerosis and diabetes mellitus. Hyperglycemia can induce oxidative stress and tissue damage through either repeated acute changes in glucose metabolism or long-term biomolecular glycation and AGE formation [110]. This can further trigger inflammation and cell proliferation contributing to the development of atherosclerosis and vascular dysfunction through the initiation of oxidation of low-density lipoproteins (LDLs) and their interaction with mononuclear cells, endothelial cells, and smooth muscle cells [111–113]. Glycation of LDLs increases their atherogenicity, while high-density lipoproteins (HDLs) have been reported to impede the glycation of LDL apolipoprotein B (apoB) [114].

In an atherosclerotic lesion, macrophages express scavenger receptors on the surface of their cell membrane to bind oxidized LDLs from blood vessel walls and to develop into foam cells. The oxidation of LDLs causes the formation of HNE-apoB adducts that contribute to the atherogenicity of LDLs and their binding capacity to scavenger receptors [110]. Additionally, the transportation of oxidized lipids in lipoprotein complexes has been suggested to play a role in the pathogenesis of atherosclerosis, those transported by LDL being associated with high risk, while those transported by HDL being indicative for protection against disease progression [115].

The LDL receptor has a high affinity to apoE which in humans exists in three isoforms: apoE2, apoE3, and apoE4, the latter being a major risk factor for cardiovascular diseases and Alzheimer's disease (Figure 1(a)). The redox status of various serum apoE isoforms determined by oxidative modification in their redox-sensitive cysteine residues has been shown to be different. The quantitative ratios of nonreduced apoE to total serum apoE from patients with apoE4/E3 were higher than those from apoE3/E3 subjects; this may be used as the disease indicator [116].

### 3.3. Diabetes Mellitus

The key roles of oxidative stress in the onset of diabetes mellitus and in the development of its complications have been demonstrated in various animal models. For example, under impaired redox balance conditions, increased Nrf2 and nitrotyrosine levels along with decreased SOD2, GPx, HO1, and endothelial nitric oxide synthase (eNOS) levels have been demonstrated in diabetic skin in mice [117]. Impairment in lipid and glucose metabolism, oxidative phosphorylation, and phospho-5′-AMP-activated protein kinase-*α*- (AMPK*α*-) mediated signaling along with the downregulation of eNOS, HO1, and sarcoplasmic reticulum calcium-ATPases 1 and 2 (SERCA 1 and SERCA 2) has been observed in diabetic rat skeletal muscle [118].

Reactive aldehydes such as HNE, when excessively produced under oxidative stress conditions, exhibit cytotoxic effects and play key roles in the pathophysiology of diabetes mellitus through the involvement in both development and progression of the disease [119]. For example, increased protein carbonyl content was observed in patients with type 2 DM associated with neuropathy [120]. Increased levels of oxidative stress biomarkers along with oxidized lipid accumulation and serum albumin glycoxidation have been reported in diabetic mice [121]. AGE-modified albumin causes diabetes-induced liver damage and impairment in the activities of proteolytic enzymes and ETC carriers. Both experimental and clinical diabetes mellitus are characterized by impaired wound healing and defect in vascular endothelial growth factor (VEGF) expression. Lipid peroxidation reactions have been shown to be involved in the pathogenesis of altered VEGF regulation and angiogenesis to stimulate wound healing in diabetic mice [122].

The high glucose concentration observed in diabetes mellitus activates the polyol (sorbitol-aldose reductase) pathway, which leads to intracellular sorbitol accumulation. The inability of sorbitol to pass through the cell membrane in insulin-independent tissues (the retina, kidney, and nervous system) causes an increase in intracellular osmotic pressure and, subsequently, cell damage. Under oxidative stress conditions, all intermediates of the polyol pathway (sorbitol, fructose, and fructose-1-phosphate) can glycate proteins leading to AGE formation, and this is implicated in microvascular complications of diabetes mellitus [123]. Interestingly, an increase in glucose and glycogen levels observed under caloric restriction conditions has been found to cause the significant decrease in the activities of the polyol pathway enzymes, along with the activation of hexokinase, glucose-6-phosphate-dehydrogenase (pentose phosphate pathway enzyme), and glucose-6-phosphatase (glycogen degradation enzyme) in both diabetic and nondiabetic rats [124]. Therefore, caloric restriction contributes to the attenuation of hyperglycemia observed in diabetes mellitus.

Also, glycoxidation of IgG by methylglyoxal generated by hydrogen peroxide has been shown to create novel epitopes and to alter IgG immunogenicity in patients with type 2 DM [125]. Through binding to their receptors, RAGEs, AGEs can greatly accelerate the progression of the disease and the development of its microvascular complications such as diabetic nephropathy, retinopathy, and neuropathy [126–128]. The AGE-RAGE axis has been implicated in cell capillary loss, capillary basement membrane thickening, increased vascular permeability, and disruption of the blood-tissue barrier, along with increased leukocyte-to-endothelial cell adhesion and neovascularization observed in experimental animal models with DM [129].

Type 2 diabetes has been characterized by the formation of glycated hemoglobin along with increased levels of serum AGEs and full-length RAGE [130, 131]. Furthermore, patients with vascular complications had a significantly higher level of the soluble form of RAGE (sRAGE), decoy AGE receptor, than those without complications, while the level of sRAGE was associated with the severity of nephropathy [132]. Patients with type 1 diabetes have been shown to demonstrate higher levels of sRAGE and endogenous secretory RAGE (esRAGE) as compared to healthy donors [133, 134].

The blockade of RAGE using the sRAGE extracellular ligand-binding domain has been demonstrated to cause wound healing and the suppression of cytokines TNF-*α* and IL-6 and matrix metalloproteinase-2, -3, and -9 expression in diabetic mice [135]. This was accompanied by increased levels of platelet-derived growth factor (PDGF) and VEGF along with the enhancement of well-vascularized granulation tissue. Impaired angiogenic response in diabetic mice was dependent on RAGE-mediated regulation, while sRAGE restored diabetes-associated impairment of angiogenic response *in vivo* [136].

The formation of AGEs has been reported to correlate with glycemic control. For example, AGE-modified serum albumin and apolipoprotein A-II levels are highest in patients with type 2 DM with poor glycemic control; in total, 19 modification sites corresponding to 11 proteins have been identified using a highly sensitive proteomic approach with the application of reverse-phase HPLC and mass spectrometry [137]. Additionally, fibrinogen and insulin-like growth factor- (IGF-) binding protein 1 are tightly connected to metabolic changes and vascular complications in patients with diabetes mellitus. The complexes of these two proteins have been shown to undergo glycoxidation, which reduces their stability and is possibly implicated in the hypercoagulation observed in type 2 DM [138].

All the abovementioned oxidative stress-induced metabolic and structural alterations may underlie the so-called “metabolic memory,” the phenomenon that consists in the development of micro- and macrovascular complications of diabetes mellitus even after improved glucose levels [139]. Early intensive glycemic control can decrease the risk of diabetic vascular complications as shown in diabetic rats, in which oxidative stress and nitric oxide levels in urine and the renal cortex soon after the establishment of good glycemic control were not different from those observed in healthy animals [140]. However, when glycemic control was delayed to 6 months, diabetic nephropathy developed in diabetic rats. Hyperglycemia induces oxidative stress, which if prolonged, causes mitochondrial dysfunction, polyol pathway activation, ALE production, AGE-RAGE axis stimulation, and subsequent diabetic vascular complications.

## 4. Conclusion

Accumulated data evidence that oxidative stress-induced excessive generation of reactive aldehydes produced through lipid peroxidation and glycoxidation reactions with consequent protein cross-linking, oligomerization, and aggregation and formation of protein oxidation adducts are implicated in aging and various chronic age-related diseases. In the present review, we focused on neurodegenerative diseases and cardiovascular disorders, complications of diabetes mellitus, and atherosclerosis, the incidence and prevalence of which increase with age. These age-related chronic diseases are becoming a major challenge for medicine and public health worldwide, because the number of subjects suffering from these increases, causing demographic changes all over the world. This dictates more investigations in the field to elucidate metabolic and structural changes that lead to alterations in cell signaling events with the involvement of ALEs and AGEs in the onset and progression of the age-associated diseases. The discovery of novel oxidative stress biomarkers and drug targets and new approaches in their clinical applications along with reconsidering health care policies are of crucial importance.

## Abbreviations

ROS: Reactive oxygen species
AOS: Antioxidant system
ETC: Electron-transportation chain
NOS: Nitric oxide synthase
ALE: Advanced lipoxidation end product
AGE: Advanced glycation end product
GSH: Glutathione
RAGE: Receptor for AGEs
PUFA: Polyunsaturated fatty acid
PL: Phospholipid
RCS: Reactive carbonyl species
MDA: Malonic dialdehyde
ACR: Acrolein
HNE: 4-Hydroxy-2-nonenal
HHE: 4-Hydroxy-hexanal
ONE: 4-Oxo-2-nonenal
CML*:*: *N*-(Carboxymethyl)-lysine
CMC: *N*-(Carboxymethyl)-cysteine
COX: Cyclooxygenase
NF-*κ*B: Nuclear factor-*κ*B
Nrf2: Nuclear factor-erythroid 2-related factor 2
TNF-*α*: Tumor necrosis factor-*α*
EGF: Epidermal growth factor
TGF-*β*: Transforming growth factor-*β*
VEGF: Vascular endothelial growth factor
IGF: Insulin-like growth factor
PDGF: Platelet-derived growth factor
MAPK: Mitogen-activated protein kinase
PTEN: Phosphatase tensin homolog deleted from chromosome 10
PINK1: PTEN-induced kinase 1
UPS: Ubiquitin-proteasome system
HSP: Heat shock protein
SOD: Superoxide dismutase
GPx: Glutathione peroxidase
GR: Glutathione reductase
ECM: Extracellular matrix
ER: Endoplasmic reticulum
UPR: Unfolded protein response
AD: Alzheimer's disease
PD: Parkinson's disease
HD: Huntington disease
ALS: Amyotrophic lateral sclerosis
LDL: Low-density lipoprotein
HDL: High-density lipoprotein
apoB: Apolipoprotein B
NMR: Naked mole-rat
RPE: Retinal pigment epithelium.
